# Supplementary material for: Leukocyte mitochondrial DNA copy number is a potential non-invasive biomarker for psoriasis
Source: PLoS One. 2022 Jun 29;17(6):e0270714. doi: 10.1371/journal.pone.0270714 (PMC9242485; doi:10.1371/journal.pone.0270714)
Supplement: Attachment — Submitted filename: Response to Reviewers (1).docx [file pone.0270714.s001.docx]

**Response to Reviewers**

Reviewer #1: In this study, these authors have investigated that mtDNA-CN is a potential biomarker for psoriasis. This is an important finding in the field of psoriasis. The manuscript is compact and well written. Below are comments aimed at improving this important study even further:

1. All data present bar graph. I recommend to refine bar graph with overlapping dots plot or dot plot (Figure 1-3).

**Our response**

Dots plot along with the bar graphs for Figure 1-3 are added in the revised manuscript (yellow highlight).

2. These authors described “the first to measure mtDNA-CN in psoriasis patients” in Abstract. I believe that this is true, however this sentence is prone to trouble for Journal (Plos One). I recommend to omit “the first”.

**Our response**

This sentence is deleted in the revised manuscript.

3. I recommend to refine detailed statistical significance in Abstract (page 2 line 12 and 14).

**Our response**

Detailed statistical significance in Abstract (page 2 line 12 and 14) is improved in the revised manuscript.

Reviewer #2: The manuscript entitled " Leukocyte mitochondrial DNA copy number is a potential non-invasive biomarker for psoriasis " describes a clinical study aimed to investigate the role of mtDMA in psoriasis. Although the experimental design and methods overall look appropriate, the manuscript requires significant revision.

Q1: Before treatment, there was a significant difference in age between the psoriasis group and the normal group. How to exclude the influence of age in this study?

**Our response**

Age of participants was included as covariate in linear regression in order to rule out confounding effect. The results showed no significant association between age and mtDNA-CN (*r^2^* = 0.014, P=0.47). This statement is included in the results of the revised manuscript (yellow highlight).

Q2: This study showed a decrease in mtDNA in the serum of patients with psoriasis and investigated the relationship between mtDNA and the duration and severity of the disease, as well as its potential use as a biomarker for psoriasis. However, the relationship between mtDNA and mitochondrial function has not been directly proved here. It is suggested that the author add other blood examination indicator related to mitochondrial function and compare them with mtDNA to further clarify the influence of mtDAN on mitochondrial function.

**Our response**

We agree with this valuable comment of the reviewer. However, previous reports have clearly shown impaired mtDNA and mitochondrial function in psoriasis (as mentioned in the introduction). Based on these previous studies, the aim of our study was to investigate changes in mtDN copy number (mtDNA-CN) in blood of psoriasis patients and the potential use of mtDNA-CN as a biomarker for psoriasis.

The suggestion of the reviewer to add other blood examination indicators related to mitochondrial function and compare them with mtDNA to further clarify the influence of mtDNA on mitochondrial function will be added as a limitation in our study, in the conclusion section of the revised manuscript (yellow highlight).

Q3: Psoriasis is a chronic immune-mediated inflammatory skin disease, which is closely related to the function of mitochondria. The authors note: “Oxidative damage to mtDNA can result in mutations and alterations in mtDNA replication and/or transcription efficiency and mtDNA-CN, which may subsequently lead to a decline in mitochondrial function with more ROS production”. Mitochondria are the main sites of ROS production, and high concentration of ROS can promote oxidative stress response, and the increase of oxidative stress and inflammation will further induce mitochondrial damage, leading to cell apoptosis. However, the author did not detect ROS and inflammation indicators, so it is suggested to add the content of this part to clarify the effects of mtDNA damage on ROS, inflammation and apoptosis, and whether mtDNA changes affect psoriasis by regulating the above pathways?

**Our response**

Since previous studies have shown a clear relationship between mtDNA-CN and increased ROS and inflammation, we focused on our main aim which is investigating the potential use of mtDNA-CN as a blood-based biomarker for psoriasis. Nevertheless, we mentioned this suggestion as a future studies in the conclusion section of the original manuscript as follows: “additional studies on the correlation of mtDNA-CN with inflammatory and oxidative stress markers can help in understanding the role of mtDNA content in the pathophysiologic mechanism(s) of psoriasis”.

Q4: Please add the code/number of ethics in the manuscript.

**Our response**

The number of ethics is added in the method section of the revised manuscript (yellow highlight): “No. 2016/496”.

Q5: Statistical analyses require clarification. Were the data normally distributed? Please indicate in the chart notes which data conform to normal distribution and test method used.

**Our response**

The normal distribution of the data of the study group was assessed by the Kolmogorov-Smirnov. This is already mentioned in the statistical analysis section of the original manuscript.

In the revised manuscript we also added the test results (P=0.2) confirming the normal distribution of data (yellow highlight).

Q6: Please provide a statement of interest.

**Our response**

The following section is added in the revised manuscript (yellow highlight)

“Competing interests: The authors declare that they have no competing interests”.
