# Supplementary material for: Leukocyte mitochondrial DNA copy number is a potential non-invasive biomarker for psoriasis
Source: PLoS One. 2022 Jun 29;17(6):e0270714. doi: 10.1371/journal.pone.0270714 (PMC9242485; doi:10.1371/journal.pone.0270714)
Supplement: Attachment — Submitted filename: second response to reviwers.docx [file pone.0270714.s002.docx]

Dear Editor,

Thanks for your valuable comments. Please find below our response:

1. The purposes and results are slightly inconsistent across the abstract, introduction, discussion, and conclusion sections, please ensure they are the same throughout the manuscript so that they are easy to understand.

**Response**

The suggested changes have been done all over the manuscript (yellow highlights).

2. Please combine the known mitochondrial information that forms the basis of the discussion section with the introduction section.

**Response**

The suggested changes have been done all over the manuscript (yellow highlights). Please note that because of these changes, some redundant references have been removed and the order of the references has been changed (yellow highlights).

3. Make the conclusion a little clearer. For limitation, please make a separate limitation section or add it to the discussion section as this needs to be described separately to the conclusion.

**Response**

The conclusion has been made clearer and the limitations section has been moved to the discussion (yellow highlights).
